# Real-world neuropsychiatric safety profile of foslevodopa/foscarbidopa infusion in advanced Parkinson's disease

**DOI:** 10.1007/s00415-026-14113-4

**Published:** 2026-09-21

**Authors:** Giulia Lazzeri, Roberta Zangaglia, Carlo Fazio, Elena Contaldi, Salvatore Bonvegna, Simone Regalbuto, Simone Malaspina, Gianni Pezzoli, Alice Jacqueline Mariangela Jelmoni, Silvia Piazza, Francesca Valentino, Luca Magistrelli, Ioannis Ugo Isaias, Antonio Pisani

**Affiliations:** 1 Parkinson Institute of Milan, ASST Gaetano Pini-CTO, 20126 Milan, Italy; 2 IRCCS Mondino Foundation, Pavia, Italy; 3 Department of Brain and Behavioral Sciences, University of Pavia, Pavia, Italy; 4 Fondazione Pezzoli Per La Malattia Di Parkinson-ETS, Milan, Italy; 5 Neurology Department, University Hospital of Würzburg, Julius Maximilian University of Würzburg, 97070 Würzburg, Germany

**Keywords:** Foslevodopa/foscarbidopa, Parkinson’s disease, Neuropsychiatric adverse events, Continuous infusion

## Abstract

**Background:**

To describe demographic, clinical, and neuropsychiatric safety outcomes in a cohort of PD patients treated with LDp/CDp infusion in a real-world setting.

**Methods:**

We retrospectively analyzed a database of 77 consecutive PD patients treated with LDp/CDp at two Italian tertiary referral centers, with a minimum 6-month follow-up. Baseline cognitive, epidemiological, and clinical variables were assessed to determine potential neuropsychiatric adverse event (NAE) risk factors.

**Results:**

Patients had long disease duration (13.8±6.4 years) and moderate-to-severe motor burden (MDS–UPDRS III: 34.4±15). Pre-existing cognitive impairment (MCI 41.6%, dementia 7.8%), prior hallucinations (26%), and impulse control disorders (27.3%) were frequent. At 6 months, mean L-dopa equivalent daily dose increased by ~300 mg (*p*<0.001). The dropout rate was 16.9% (13 patients), mainly due to AEs (38.5%), bridging to DBS (23%), or device intolerance (15.4%). New-onset NAEs emerged in 17 patients (22.1%) at a median of 30 days, predominantly hallucinations (41.2%) and psychosis (29.4%). Most events were efficiently managed via careful infusion rate adjustments and low-dose antipsychotics, achieving resolution or improvement in 82% of cases. Three patients (18%) discontinued therapy due to severe NAEs. Multivariable regression analysis confirmed baseline Frontal Assessment Battery (FAB) score as the primary independent predictor of NAE development (OR 0.707, *p*=0.010).

**Conclusions:**

LDp/CDp infusion is well-tolerated. Although NAEs were more frequent than in pivotal trials, they were largely manageable. Frontal executive dysfunction emerged as a significant risk factor, suggesting specific frontal screening should be routine in candidacy evaluation for cognitively frail patients. Preventive strategies to reduce risk are proposed and discussed.

## Introduction

Parkinson’s disease (PD) is a progressive neurodegenerative disorder characterized by a continuum of motor and non-motor manifestations, with oral levodopa representing the cornerstone of symptomatic therapy. As the disease advances, the short plasma half-life and variable bioavailability of orally administered levodopa, related to its restricted absorption in the proximal small intestine, gastrointestinal dysfunction, peripheral metabolism, and interference from dietary amino acids, may lead to fluctuations in levodopa availability and intermittent dopaminergic stimulation. In parallel, the progressive loss of striatal dopaminergic buffering capacity increases the clinical impact of these pharmacokinetic fluctuations, contributing to motor and non-motor fluctuations [1, 2]. Continuous dopaminergic delivery can effectively overcome these limitations by providing more stable dopaminergic stimulation [3, 4].

Device-aided therapies, including levodopa–carbidopa intestinal gel (LCIG), levodopa–carbidopa–entacapone intestinal gel, continuous subcutaneous apomorphine infusion (CSAI) and, more recently, foslevodopa/foscarbidopa subcutaneous infusion (LDp/CDp), have been developed to implement this delivery approach and to mitigate motor fluctuations [5, 6].

LDp/CDp is a prodrug formulation of levodopa/carbidopa delivered via continuous subcutaneous infusion, providing pharmacokinetic advantages over oral administration [7]. Unlike LCIG, the distinct pharmacokinetics associated with subcutaneous delivery and the absence of intestinal absorption issues may alter the safety profile, thereby necessitating specific real-world investigation.

While pivotal clinical trials have demonstrated its efficacy and tolerability [8, 9], patients enrolled in these studies were highly selected, excluding those with significant cognitive impairment or severe active psychiatric comorbidities. Consequently, real-world data regarding neuropsychiatric outcomes remain limited. This knowledge gap is critical, because neuropsychiatric manifestations, including hallucinations, psychosis, and impulse control disorders (ICDs), are common in advanced PD and may be influenced by dopaminergic therapies, including levodopa-based infusions such as LCIG and dopamine agonists such as CSAI [10–12]. Given the rapid clinical adoption of this therapy and its use in increasingly heterogeneous patient groups, real-world evidence is needed to inform risk stratification, guide safe titration, and clarify the clinical evolution of these events.

To address this gap, we conducted a retrospective real-world study of 77 consecutive patients with advanced PD treated with continuous subcutaneous LDp/CDp infusion at two tertiary referral centers. The primary objective was to characterize the neuropsychiatric safety profile of LDp/CDp in routine clinical practice and to identify demographic, cognitive, and treatment-related factors associated with the development of neuropsychiatric adverse events (NAEs). Finally, we describe the management strategies adopted and the clinical outcomes of NAEs over follow-up.

## Materials and methods

We conducted a retrospective, descriptive, observational study using prospectively collected clinical data from consecutive patients with advanced PD who initiated treatment with continuous infusion of LDp/CDp at two Italian tertiary referral centers. Patients who remained on LDp/CDp treatment were required to have at least 6 months of documented clinical follow-up, whereas patients who discontinued treatment before 6 months were retained in the safety analysis, with all available clinical data up to treatment discontinuation included.

### Data collection

Demographic and epidemiological variables included sex, age at treatment (AaT), age at disease onset (AaO), disease duration (DD), and body mass index (BMI).

Inclusion criteria were the following: (i) a diagnosis of idiopathic PD according to current criteria [13]; (ii) initiation of LDp/CDp infusion with either at least 6 months of documented follow-up or treatment discontinuation before 6 months; and (iii) availability of complete motor and cognitive assessment at baseline and at the last known follow-up: MDS Unified Parkinson’s Disease Rating Scale (MDS–UPDRS) parts III–IV, Mini-Mental State Examination (MMSE) [14], the Montreal Cognitive Assessment (MoCA) [15], and the Frontal Assessment Battery (FAB) [16]. MDS–UPDRS III was assessed during clinically evident wearing-off periods as part of routine clinical practice, although the timing relative to the previous dopaminergic dose was not standardized. Neuropsychological assessments were performed during the usual treated state to minimize the potential confounding effect of motor fluctuations on cognitive performance. Cognitive status [normal cognition, mild cognitive impairment (PD–MCI), Parkinson’s disease dementia (PDD)] was assigned according to the last known MDS criteria [17, 18].

History collection focused on the presence of neuropsychiatric comorbidities, such as psychosis, hallucinations, and ICD. Treatment-related data included infusion parameters, concomitant use of antipsychotics or anti-acetylcholinesterase (anti-AChE) and modifications of dopaminergic therapy, including concomitant use of dopamine agonists (DA), catechol-o-methyltransferase inhibitors (COMT) or monoamine oxidase-B (MAO-B) inhibitors. The Levodopa Equivalent Daily Dose (LEDD) was calculated according to standard conversion factors [19–21].

### Safety assessment

NAEs were ascertained through a systematic review of medical records, where neuropsychiatric status is routinely queried at each follow-up visit. Each AE was categorized according to type (hallucinations, psychosis, ICD, punding, dopamine dysregulation syndrome [DDS], or other) and was further characterized in terms of clinical outcome (stable, improved, refractory or requiring therapy change), management and resolution status.

### Study design and analysis

Continuous variables are reported as means ± standard deviation and ranges. Categorical variables are expressed as counts and percentages. In order to identify risk factors for such events, the cohort was subdivided into two groups on the basis of NAEs development. Data normality was assessed using the Shapiro–Wilk test. Between-group comparisons were performed using the unpaired Student’s *t* test or the Mann–Whitney *U* test for continuous variables, depending on data normality. Categorical variables were compared using the *χ*^*2*^ test or Fisher’s exact test, as appropriate. For paired samples, the paired Student’s *t* test or Wilcoxon signed-rank test was used for continuous variables, and the McNemar test for categorical variables. Spearman correlation analysis was used due to the non-normal distribution of the data. Partial Spearman correlations, adjusted for demographic and baseline clinical characteristics, were calculated to control for potential confounders. Binomial logistic regression models were used to identify predictive factors of NAEs development. Continuous variables were standardized (z-scores) before logistic regression to facilitate comparison of effect sizes. To prevent overfitting given the limited number of events (*n=*17), we avoided including all predictors in a single model; instead, we performed separate multivariable logistic regression models, each including a maximum of two predictors (e.g., FAB score plus one therapeutic variable). Assumptions for binomial logistic regression were assessed, including linearity of continuous predictors with the logit of the outcome, absence of multicollinearity, and lack of influential outliers. Survival analysis was performed using Kaplan–Meier curves, stratifying patients based on literature-accepted cutoffs for FAB (< 13.4) [16] and MoCA (< 17.36) [15] to assess the cumulative incidence of NAEs. Differences between survival curves were evaluated using the log-rank test. Statistical significance was set at *p*<0.05. All statistical analyses were performed using IBM SPSS Statistics software version 25.

### Ethics

The study was conducted in accordance with the Declaration of Helsinki. The study protocol was approved by the relevant Ethics Committees at the participating institutions: for data collected at ASST Gaetano Pini-CTO (Milan), approval was granted by the Comitato Etico Territoriale Lombardia 3 (protocol no. 3499/23); for patients enrolled at IRCCS Mondino Foundation (Pavia), approval was granted by the Comitato Etico Territoriale Lombardia 6 (approval no. 2025–3.11/303). Written informed consent was obtained from all participants at treatment initiation.

## Results

### Epidemiologic and clinical characteristics

Patients initiating LDp/CDp infusion presented with a long disease history and advanced clinical profile with moderate motor burden and fluctuation degree (MDS–UPDRS III: 34.4 ± 15, MDS–UPDRS IV: 8.3 ± 3.5). The mean age at therapy initiation was 66.3 ± 9 years, with a mean age at disease onset of 52.5 ± 10.6 years, corresponding to a disease duration of approximately 14 years and a mean latency of 7 years between onset and the appearance of motor fluctuations. The average body mass index (BMI) at baseline was 23.6 ± 4.3, and the cohort was balanced by sex (37 females, 40 males).

Cognitive screening highlighted a profile consistent with mild cognitive impairment. The mean MMSE score was 26 ± 3.6, indicating globally preserved orientation and basic cognitive abilities, whereas the mean MoCA was 20.4 ± 4.5, reflecting more subtle deficits in attention, executive function, and visuospatial skills. The FAB averaged 13.6 ± 3.6, suggesting mild but detectable impairments in executive domains. (Table 1).

**Table 1:** Baseline epidemiological and clinical characteristics of 77 patients treated with LDp/CDp infusion

	Value (mean ± SD/n)	Range/details
Sex (Female/Male)	37/40	-
Age at treatment (years)	66.3 ± 9	35–80
Age at disease onset (years)	52.5 ± 10.6	24–71
Disease duration (years)	13.83 ± 6.37	3–35
MDS–UPDRS III	34.4 ± 15	7–81
MDS–UPDRS IV	8.3 ± 3.5	1–19
BMI	23.56 ± 4.31	13.96–31.2
MMSEc	26 ± 3.6	12–30
MoCAc	20.4 ± 4.5	7.4–28.1
FABc	13.6 ± 3.6	5.9–18.1
History of PD–MCI/PDD	38 (49%)	32 PD–MCI/6 PDD
History of hallucinations/psychosis	20 (26%)	17 hallucinations/3 psychosis
History of ICD	21 (27%)	18 previous/3 active
Total LEDD (mg)	*1104.5* ± *366.6*	-

*SD* Standard Deviation, *MDS–UPDRS* MDS Unified Parkinson's Disease Rating Scale, *BMI* Body Mass Index, *MMSE* Mini-Mental State Examination, *MoCA* Montreal Cognitive Assessment, *FAB* Frontal Assessment Battery, c age- and education-corrected scores, *PD–MCI* Parkinson’s Disease–Mild Cognitive Impairment, *PDD* Parkinson’s Disease Dementia, *ICD* Impulse Control Disorder

Clinically, 49% of the cohort met criteria for cognitive decline (32 PD–MCI, 6 PDD). 26% of patients reported a history of levodopa/DA-induced hallucinations (*n =*17) or psychosis (*n=*3), while 27% (*n=*21) reported ICD, either previously resolved (*n=*18) or active but stable at implantation (*n=*3).

### Therapeutic approach

At baseline, the mean total LEDD was 1104 ± 366 mg. Dopamine agonists accounted for approximately 186 mg of the total LEDD and were used in 56% of patients, while MAO-B inhibitors and COMT inhibitors were used in 34% and 53% of patients, respectively. Before the infusion, amantadine was administered sporadically (10 patients, 13%), whereas anticholinergics were not prescribed. Antipsychotic medications were already prescribed in 7 patients (9%), while anti-AChE drugs were rare (5%). Before implantation, 4 patients were already using other device-aided therapies (2 CSAI, 2 LCIG), while 4 patients with Deep Brain Stimulation (DBS) were deemed eligible for add-on therapy with LDp/CDp, with a mean interval of 6.0 ± 3.4 years between DBS implantation and initiation of LDp/CDp infusion. Furthermore, DBS settings were stable at the time of LDp/CDp initiation and throughout follow-up.

After 6 months from initiation of LDp/CDp infusion, the total LEDD increased to 1325.6 ± 520.2 mg, with a significant reduction in the DA LEDD and the use of COMT and MAO-B inhibitors, while amantadine use did not diverge from baseline (Table 2). Given its limited use in our cohort and frequent administration at reduced doses, it was selectively maintained for dyskinesia control when the perceived benefit was considered to outweigh its potential neuropsychiatric burden, resulting in an overall acceptable risk–benefit profile.

**Table 2:** Therapy data and clinical scales at baseline and follow-up

	Baseline	Follow-up	p
Total LEDD (mg)	1104.5 ± 366.6	1325.6 ± 520.2	**.001**
DA (%)	56	32.5	**<.001**
DA LEDD (mg)	154.16 ± 101.1	118.8 ± 79.9	**.011**
MAO-B inhibitors (%)	34	18	**.002**
COMT inhibitors (%)	53	19.5	**<.001**
Amantadine (%)	13	10.3	ns
Anticholinergics (%)	0	0	ns
Antipsychotics (%)	9	27.3	**<.001**
Anti-AChE (%)	5	10	**<.001**
Day Infusion Rate (mL/h)	0.29 ± 0.1	0.33 ± 0.11	**<.001**
Night Infusion Rate (mL/h)	0.17 ± 0.04	0.20 ± 0.09	**<.001**
16 h infusion (%)	1.3	7.8	**<.001**
MDS–UPDRS III	34.4 ± 15	27.0 ± 13.0	**<.001**
MDS–UPDRS IV	8.3 ± 3.5	5.25 ± 2.83	**<.001**

*SD* Standard Deviation, *LEDD* Levodopa Equivalent Daily Dose, *DA* Dopamine agonist, *MAO-B* Monoamine oxidase-B, *COMT* Catechol-O-methyltransferase, *Anti-AChE* Anti-acetylcholinesterase, *ns* not significant, *MDS–UPDRS* MDS–Unified Parkinson's Disease Rating Scale. Wilcoxon or McNemar tests were used

The p value is indicated in [bold] when statistically significant

Infusion rates were optimized over the 6 months, increasing from a mean baseline daytime rate of 0.29 mL/h to 0.33 mL/h, and night-time rate from 0.17 mL/h to 0.20 mL/h. In a minority of cases (increase from 1.3% to 7.8%), patients were switched to a 16-h infusion regimen, primarily as a strategy to manage nocturnal adverse events or improve tolerability. At follow-up, antipsychotic use increased substantially, with 14 patients initiating treatment, while anti-AChE prescriptions also increased to address emerging cognitive symptoms (Table 2).

Motor efficacy was confirmed by a significant reduction in MDS–UPDRS IV scores (from 8.3 to 5.25, *p* < 0.001) and MDS–UPDRS III scores (from 34.4 to 27.0, *p* < 0.001; Table 2). Although MDS–UPDRS Part IV items were not systematically available due to the retrospective design, clinical assessments during follow-up indicated that several patients remained dyskinetic, while some experienced residual OFF periods, particularly after meals.

### Neuropsychiatric adverse events

Eight patients (10.3%) had active NAEs with oral therapy before the infusion, mostly mild visual hallucinations, ICD, delusional ideas, or DDS; all of them were well-controlled with antipsychotic therapy (low doses of either quetiapine or clozapine).

New-onset NAEs occurred in 17 patients (22%) and were generally stabilized or improved with therapeutic adjustments. Mean time from implantation to NAEs development was 81.7 ± 134 days (range 1–450). Interestingly, eight patients developed NAEs within the first 2 weeks of LDp/CDp initiation. Compared with patients developing NAEs later during follow-up, no consistent differences were observed in terms of disease characteristics, previous neuropsychiatric history, or pharmacological treatment. Among early-onset cases, psychotic and confusional symptoms, including hallucinations and delusions, represented the most frequent clinical presentation. (Table 3).

**Table 3:** Neuropsychiatric adverse events (NAEs) observed during LDp/CDp infusion

	*n* (%)	Median onset (d)	Management	Outcome/Details
None reported	52 (68)	–	–	
Pre-existing NAEs	8 (10)	–	Antipsychotics, DA reduction, AChE introduction	3 hallucinations, 3 ICD, 1 DDS, 1 delusional ideas. All stable
New onset NAEs	17 (22)	30 (1–450)		
Hallucinations	7 (9)	14 (1–365)	Antipsychotics	Resolution in 6 cases, 1 drop-out
Psychosis	5 (6)	30 (3–180)	Antipsychotics, dose modulation, night-time dose suspension	Resolution in 4 cases, 1 drop-out
Punding	1 (1.3)	450	Dose reduction, AChE introduction	Resolution
Behavioural change	1 (1.3)	30	Antipsychotics	Improvement
DDS with refractory agitation	1 (1.3)	120	Antipsychotics, dose modulation, DA interruption	Already present mild DDS, drop-out
Psychomotor agitation (night)	2 (3)	3.5 (2–5)	Night-time dose modulation	Resolution

*NAEs* neuropsychiatric adverse events, *ICD* Impulse Control Disorder, *DDS* Dopamine Dysregulation Syndrome, *Da* Dopamine agonist, *AChE* Acetylcholinesterase inhibitors. Each patient was counted once according to the predominant NAEs phenotype

Newly emerging hallucinations, mostly distressing and arising at night, were observed in 7 cases, and were managed with dose adjustment (*n=*4), low-dose antipsychotics (*n=*2) or DA suspension (*n=*1), leading to resolution of symptoms in the majority of cases. Psychosis or delusional ideas were described in 5 patients, with one severe episode requiring emergency care and improved after introduction of therapy with quetiapine. Psychosis was generally easily manageable and well responsive to a combination of antipsychotic drug introduction and dosing adjustment. Punding was observed in a single patient and resolved following dose adjustment. In addition, one patient exhibited behavioral changes characterized by aggressiveness, while two other patients experienced acute psychomotor agitation during the first few days of titration that did not require any therapeutic action. Worsening of previously mild DDS, followed by refractory agitation, occurred in one patient with pre-existing PDD after transition to LDp/CDp monotherapy and led to discontinuation of the infusion.

In order to identify risk factors for the development of neuropsychiatric complications, the cohort was dichotomized into two groups depending on NAEs development (no-NAEs vs NAEs). Table 4 summarizes the comparison between groups.

**Table 4:** Comparison between two subgroups in our cohort, divided according to development of NAEs (no-NAEs vs NAEs)

	no-NAEs (*n=*60)	NAEs (*n=*17)	*p* value
Age at treatment, years (mean ± SD)	66.58 ± 8.08	65.35 ± 12.11	ns
Age at onset, years (mean ± SD)	52.52 ± 9.89	52.59 ± 13.36	ns
BMI, kg/m^2^ (mean ± SD)	23.68 ± 4.10	23.14 ± 5.13	ns
Sex (female/male)	29/31	8/9	ns
Disease duration, years (mean ± SD)	14.12 ± 6.56	12.82 ± 5.71	ns
MDS–UPDRS III BL, points (mean ± SD)	34.50 ± 15.37	34.24 ± 14.21	ns
MDS–UPDRS IV BL, points (mean ± SD)	8.40 ± 3.58	8.06 ± 3.27	ns
MoCAc, points (mean ± SD)	21.20 ± 3.77	17.46 ± 6.21	**0.036**
MMSEc, points (mean ± SD)	26.60 ± 2.73	24.10 ± 4.61	**0.023**
FABc, points (mean ± SD)	14.39 ± 3.25	11.48 ± 3.70	**0.009**
Cognitive status (normal/PD–MCI/PDD)	35/23/2	4/9/4	**0.002**
Psychiatric history (yes/no)	12/48	8/9	**0.033**
ICD history (yes/no)	16/44	5/12	ns
LEDD BL, mg (mean ± SD)	1072.55 ± 364.08	1217.19 ± 363.96	ns
LEDD DA BL, mg (mean ± SD)	172.17 ± 187.73	251.25 ± 397.72	ns
DA users BL (yes/no)	35/25	8/9	ns
COMT users BL (yes/no)	32/28	9/8	ns
Day infusion rate BL, ml/h (mean ± SD)	0.28 ± 0.09	0.34 ± 0.10	**0.027**
Night infusion rate BL, ml/h (mean ± SD)	0.16 ± 0.03	0.19 ± 0.05	**0.017**
LEDD LDp/CDp BL, mg (mean ± SD)	972.17 ± 266.35	1192.00 ± 328.16	**0.017**
Day infusion rate FU, ml/h (mean ± SD)	0.32 ± 0.10	0.36 ± 0.10	ns
Night infusion rate FU, ml/h (mean ± SD)	0.20 ± 0.07	0.19 ± 0.14	ns
LEDD LDp/CDp FU, mg (mean ± SD)	1139.35 ± 346.90	1238.40 ± 375.96	ns
Total LEDD FU, mg (mean ± SD)	1269.43 ± 457.87	1529.19 ± 681.13	ns
Duration of FU, months (mean ± SD)	7.38 ± 4.35	9.53 ± 4.89	ns
Delta MDS–UPDRS III, points (mean ± SD)	–7.77 ± 8.70	–7.25 ± 10.54	ns
Delta MDS–UPDRS IV, points (mean ± SD)	–3.49 ± 2.34	–1.81 ± 2.29	**0.036**
Delta LDp/CDp, mg (mean ± SD)	161.79 ± 230.01	46.40 ± 251.60	**0.039**

*BL* Baseline, *FU* Follow-up, *SD* Standard Deviation, *ns* not significant, *BMI* Body Mass Index, *MDS*–*UPDRS* MDS Unified Parkinson's Disease Rating Scale, *MoCA* Montreal Cognitive Assessment, *MMSE* Mini-Mental State Examination, *FAB* Frontal Assessment Battery, *PD–MCI* Parkinson’s Disease–Mild Cognitive Impairment, *PDD* Parkinson’s Disease Dementia, *ICD* Impulse Control Disorder, *LEDD* Levodopa Equivalent Daily Dose, *DA* Dopamine agonist, *COMT* Catechol-O-methyltransferase

The p value is indicated in [bold] when statistically significant

**Fig. 1:**
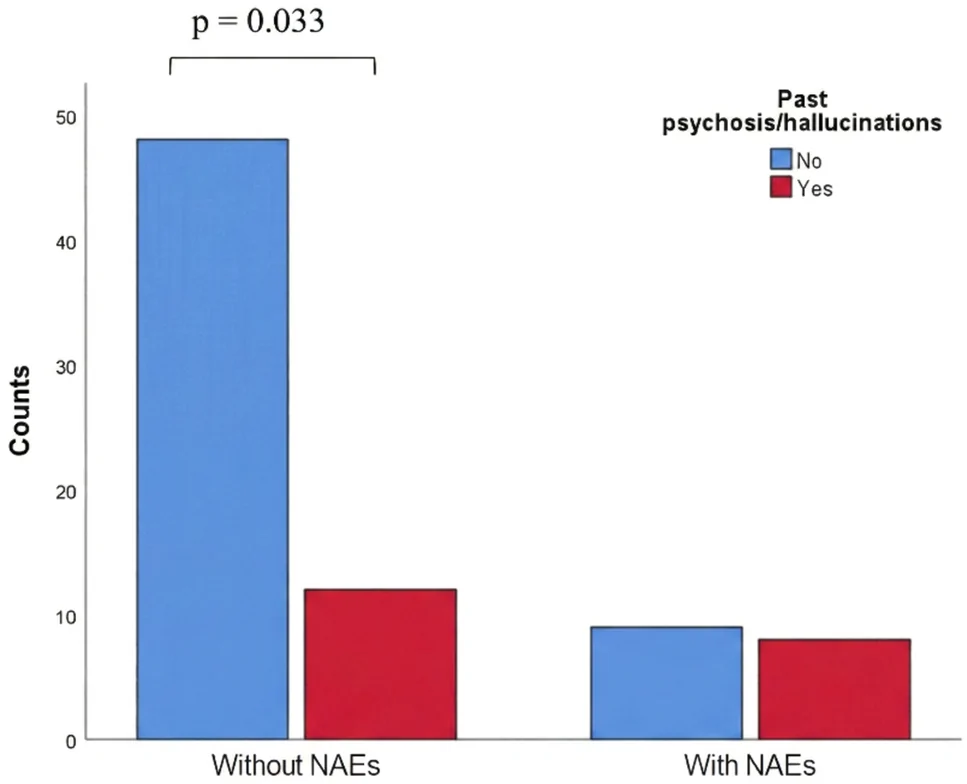
Distribution of previous psychosis or hallucinations among patients with and without new-onset neuropsychiatric adverse events (NAEs)

While no demographic or clinical differences emerged, patients who developed NAEs showed significantly worse baseline cognitive performance, with lower MoCAc, MMSEc, and FABc scores. Cognitive status distribution also differed between groups, with a higher proportion of PD–MCI and PDD among NAEs patients. A history of psychiatric disorders was more common in the NAEs subgroup (Fig. 1). Notably, the NAEs group had higher baseline infusion rates (both diurnal and nocturnal) and higher LDp/CDp LEDD at initiation. At follow-up, MDS–UPDRS IV scores improved significantly in both the no-NAEs group (8.40 ± 3.58 to 5.00 ± 2.75, *p* < 0.001) and the NAEs group (8.06 ± 3.27 to 6.13 ± 3.05, *p =* 0.012), although the magnitude of improvement was significantly lower in the NAEs group (Δ MDS–UPDRS IV: −1.81 vs −3.49, *p =* 0.036). The NAEs group also showed a lower increase in LDp/CDp dosage (Δ LDp/CDp: 46.4 vs 161.8 mg, *p =* 0.039), while total LEDD remained higher in the NAEs group (1529.2 vs 1269.4 mg), although this difference was not statistically significant.

Spearman univariate correlations highlighted significant positive correlations with day infusion rate (*r =* 0.253, *p =* 0.026), night infusion rate (*r =* 0.273, *p =* 0.016), cognitive status (*r =* 0.367, *p =* 0.001), history of psychosis and/or hallucinations (*r =* 0.255, *p =* 0.026), while a single strong negative correlation was found with FABc (*r =* − 0.310, *p =* 0.008).

To verify the consistency of these findings, we performed multivariable regression analyses testing different combinations of therapeutic variables alongside cognitive screening measures.

First, a model including daytime infusion rate confirmed that both lower FAB scores (*p =* 0.004) and higher daytime rates (*p =* 0.022) were significantly associated with NAEs. Subsequently, we tested the total daily dose of LDp/CDp (LEDD), finding that while higher baseline doses increased the risk (OR 2.05, 95% CI 1.11–3.78, *p =* 0.022), the predictive value of the FAB score remained statistically significant (OR 0.77, 95% CI 0.19–0.80, *p =* 0.010). The final model focused on the night-time infusion rate, which was supposed to be clinically critical for nocturnal hallucinations. In this model, night-time infusion rate emerged as a strong predictor (OR 2.59, 95% CI 1.26–5.36, *p =* 0.010) and FAB score maintained its independent predictive power (OR 0.748, 95% CI 0.60–0.93, *p =* 0.008) across all models, demonstrating superior robustness compared to other cognitive scales.

**Fig. 2:**
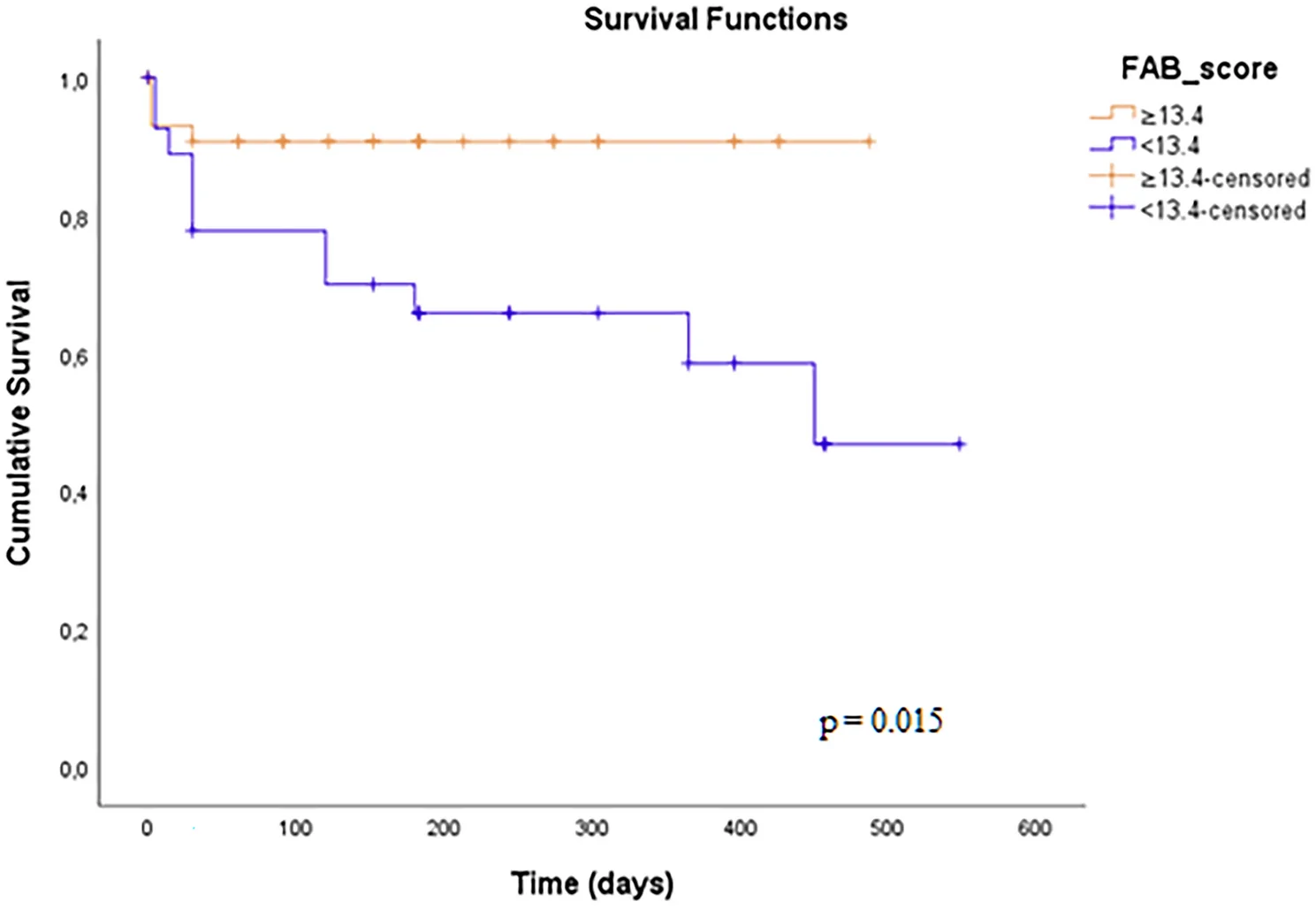
Kaplan–Meier survival curves showing the cumulative incidence of new-onset neuropsychiatric adverse events stratified by baseline FAB performance (< 13.4 vs ≥13.4)

**Fig. 3:**
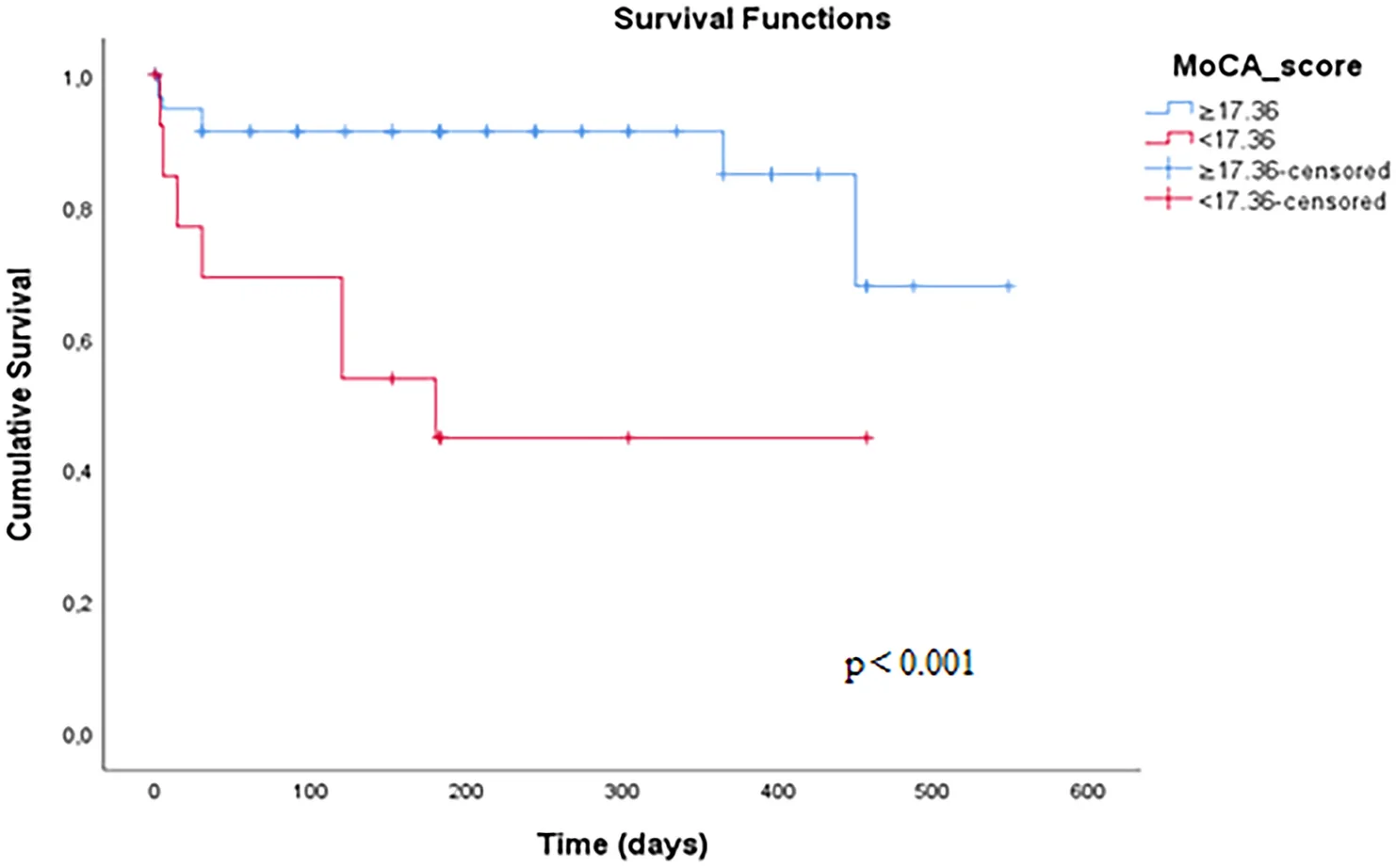
Kaplan–Meier curves illustrating the cumulative incidence of new-onset neuropsychiatric adverse events stratified by baseline MoCA impairment (< 17.36 vs ≥17.36)

Based on literature-accepted cutoffs for FAB [16] and MoCA [15], we obtained Kaplan–Meier curves showing a markedly higher cumulative incidence of new-onset NAEs in patients with pathological baseline scores compared with those above the cognitive thresholds (Figs. 2, 3).

### Causes of discontinuations

Thirteen patients (16.9%) discontinued treatment during the follow-up period, with a mean time from implantation of 85.5 days ± 73.4 (median 87 days, IQR: 16.5–165). The most frequent causes are shown in Table 5.

**Table 5:** Drop-out causes (frequency is relative to total of drop-outs)

Drop-out causes	n (%)	Details
Adverse events	5 (38.5)	3 Neuropsychiatric, 2 cutaneous (nodules)
Bridging therapy	3 (23)	Planned switch to DBS
Ineffectiveness	1 (7.7)	Subjective ineffectiveness
Intolerance of device	2 (15.4)	Rejection of the infuser; probably inconsistent expectations
Polyneuropathy	1 (7.7)	Mild axonal length-dependent neuropathy in lower limbs, asymptomatic. Discontinuation warranted to prevent worsening
Miscellaneous	1 (7.7)	Pneumonia and hospitalization leading to discontinuation

The causes for withdrawal were heterogeneous. Adverse events accounted for the largest proportion of dropouts (*n =* 5, 38.5% of discontinuations), including three neuropsychiatric cases (severe psychosis, hallucinations, and DDS) and two cases of severe dermatological complications, specifically subcutaneous nodules or infusion site reactions. Other reasons for discontinuation included bridging to DBS (*n =* 3, 23%), device intolerance or subjective lack of benefit (*n =* 3, 23%), and unrelated medical issues (*n =* 1).

Notably, one patient developed an axonal, symmetric, length-dependent lower-limb polyneuropathy on control nerve conduction studies at 6 months, leading to premature discontinuation to prevent a negative evolution.

## Discussion

This multicenter retrospective study provides real-world evidence on the neuropsychiatric safety profile of LDp/CDp in a cohort of 77 patients with advanced PD. Our findings indicate that LDp/CDp is generally well-tolerated in routine clinical practice, with most NAEs being mild, transient, and manageable through individualized dose adjustments and targeted pharmacological interventions. The treatment demonstrated an overall favorable safety profile and low discontinuation rate within the first 6 months of treatment, with excellent effectiveness in reducing motor fluctuations.

In our cohort, new-onset NAEs occurred in approximately one-fifth of patients (22.1%). This rate is slightly higher than the 15–17% reported in pivotal phase III trials [8, 9], likely reflecting the unselected nature of our population, which included patients with cognitive impairment and psychiatric comorbidities often excluded from regulatory studies. Furthermore, the complexity of our real-world cohort is highlighted by the inclusion of patients transitioning from other device-aided therapies (CSAI and LCIG) and those receiving LDp/CDp as an add-on to DBS.

When contextualized within the broader literature on device-aided therapies, our findings align with the extensive knowledge accumulated on LCIG [11, 22–24]. In real-life studies on LCIG, hallucinations ranged from 0.7% [23] to 13% [22]. In a large Italian series of patients treated with LCIG, new onset and/or worsening of hallucinations and/or confusion, reported after initiation of infusion therapy, were a cause of withdrawal (7.2%) [24]. In our cohort, only 3 patients interrupted the infusion due to NAEs.

Notably, our observed NAEs rate is significantly lower than the high rate (55%) recently reported by Brohée et al. in a smaller case series [25]. Similarly, a very recent real-world study by Desjardins et al. reported a much higher rate of neuropsychiatric worsening (47.2%) [26]. However, this discrepancy likely reflects differences in pharmacological management: while COMT inhibitor use was identified as a strong predictor of worsening in their cohort (OR 9.0), our patients underwent a significant reduction in concomitant medications, which may have played a protective role. In the series proposed by Brohée et al., the vast majority of patients (80%) had a history of hallucinations or delirium before infusion, whereas only one quarter of patients in this study had comparable antecedents. Moreover, dosing strategies differed considerably, with higher and less differentiated day–night infusion rates reported in cohorts experiencing more severe psychiatric complications.

Indeed, a critical determinant of tolerability appears to be the infusion dosing strategy. In this study, the total daily dose of LDp/CDp was significantly associated with NAEs. The mean maintenance flow rates were conservative, averaging 0.33 mL/h during the day and 0.20 mL/h at night (mean infusion-specific LEDD ~1200 mg). This conservative approach contrasts not only with the pivotal trials but also with other recent real-world data. For instance, a recent case series reported severe neuropsychiatric effects in patients maintained on high rates (approx. 0.60 mL/h day/night) [25]. Similarly, another recent real-world study reported a mean base hourly rate of 0.39 mL/h [27], which was significantly higher than the theoretically recommended calculation. Low flow rates likely mitigated nocturnal hallucinations and agitation, which represented the most frequent and temporally early NAEs.

While pivotal trials for LDp/CDp largely excluded patients with significant cognitive impairment (MMSE < 24), recent real-world data included 40% of patients with a history of cognitive impairment and 62% with a history of psychosis [27]. Notably, despite this high prevalence of psychiatric comorbidities, the authors found that only a small minority of patients discontinued due to psychosis [27]. This supports our finding that LDp/CDp is viable even in patients with a psychiatric history, provided that titration is careful and monitoring is close. Another distinctive feature of our cohort was its relatively balanced sex distribution (male/female ratio 1.08), despite the male predominance generally reported in PD. This is noteworthy given evidence that the male-to-female ratio increases with age in PD [28], although the relatively small sample size and tertiary-center setting may have contributed to this finding.

Of note, our cohort also included patients with active NAEs at the moment of implantation, who were deemed stable enough to be good candidates. Among this subset of patients, NAEs did not worsen significantly, and no discontinuation occurred, providing evidence that LDp/CDp could be a safe option also in this population. This observation is consistent with Desjardins et al., who found that patients with a history of hallucinations could be safely treated if managed proactively with clozapine [26]. In our experience, personalized preventive measures taken during drug titration, which involves a combination of lower possible night-time infusion (usually around 0.15 ml/h) and preventive antipsychotic/anti-AChE introduction, explained this favorable outcome.

Our data, derived from a larger sample, suggest that while NAEs are a tangible risk, they are not inevitable. Our findings align closely with the recent series by Toś et al. [29], who reported a 25% incidence of psychotic symptoms, describing a specific phenotype of "atypical" hallucinations time-locked to the initiation of therapy. Thus, the safety profile of LDp/CDp remains acceptable for most advanced candidates, provided that risk factors are adequately stratified.

Notably, our analysis identified baseline frontal executive dysfunction, assessed via the FAB, as a significant independent predictor of NAEs. At the same time, global screening with the MMSE performed poorly as a predictor in our model. Therefore, executive-focused assessments appear superior for risk stratification when evaluating candidacy for continuous infusion therapies.

The relationship between neuropsychiatric tolerability and motor benefit also warrants attention. Patients who developed NAEs showed a smaller reduction in MDS–UPDRS IV scores and a lower increase in LDp/CDp dosage over follow-up, suggesting that psychiatric vulnerability may limit the extent of motor optimization achievable with infusion therapy. This phenomenon highlights the clinical trade-off between motor control and neuropsychiatric safety in cognitively frail patients and underscores the importance of individualized therapeutic goals.

Interestingly, the management of ICDs in our study mirrors the beneficial effects reported in LCIG real-world studies [30, 31]. The transition to continuous infusion allowed for a significant reduction in oral dopamine agonists, a class of drugs strongly associated with ICDs. As widely documented in LCIG literature, the "de-priming" effect obtained by tapering oral agonists while maintaining stable levodopa levels can resolve or improve pre-existing compulsive behaviors [32]. Consistently, in our cohort, ICDs were manageable and rarely led to discontinuation, suggesting that LDp/CDp, like LCIG, facilitates a safer pharmacological environment for patients prone to behavioral addictions.

The vast majority of NAEs in our cohort were mild and manageable. We found that reducing the night-time infusion rate was a highly effective first-line strategy. This strategy is supported by the hypothesis that continuous 24-h stimulation may lead to mesolimbic hyperactivation and receptor desensitization [29]. This approach addresses the continuous dopaminergic stimulation that, while beneficial for motor symptoms, may disrupt sleep architecture and precipitate nocturnal hallucinations in patients with increased neuropsychiatric susceptibility. Clinically, this strategy was also reported in another Italian cohort [33], in which stopping or reducing nocturnal infusion was a necessary and effective measure to manage hallucinations and delusions in their pragmatic cohort. In cases where dose reduction alone was insufficient, the addition of low-dose atypical antipsychotics (e.g., quetiapine) proved effective, preventing discontinuation in most cases. Overall, antipsychotic use increased during follow-up, reflecting both the treatment of emerging neuropsychiatric complications and, in selected cognitively vulnerable patients, the proactive use of low-dose antipsychotics to mitigate the risk of NAEs. It has also been advocated that inpatient initiation may help mitigate these risks [27], a practice we also followed and may be recommended for at-risk patients.

Another divergent point, not yet adequately explored, is the concomitant use of other dopaminergic medications. While previous studies [27] usually described high rates of MAO-B inhibitors/COMT-I and LDp/CDp simultaneous use, our cohort showed a significant simplification of these adjunctive therapies at follow-up (MAO-B inhibitors reduced to 18% and COMT inhibitors to 19.5%), with only DA being maintained in approximately one-third of patients. This trend towards simplification is strongly supported by a recent post hoc analysis of registrational trials, suggesting that monotherapy may be as efficacious and safe as polytherapy [34]. Our lower discontinuation rate (16.9%) suggests that a combination of inpatient care, slow titration strategy, personalized approach with simplification of oral therapy, and careful adjustment of night-time dose may favor long-term retention and psychiatric tolerability even in frail patients.

This study has several limitations. Its retrospective, descriptive, and uncontrolled design precludes causal inference. A minimum follow-up period of 6 months was considered appropriate to allow sufficient time to characterize neuropsychiatric adverse events in patients who remained on treatment. Nonetheless, patients with incomplete follow-up for other reasons may have been under-represented, potentially introducing selection bias. Moreover, the heterogeneous duration of follow-up may have influenced the detection of later-emerging neuropsychiatric adverse events (median 10 months in the NAEs group vs 6 months in the no-NAEs group, *p =* 0.097). The relatively small sample size and recruitment from two tertiary centers may limit generalizability, particularly to earlier disease stages. Data collection relied on routine clinical practice, with potential missing data and lack of standardized neuropsychiatric assessments. Furthermore, MDS–UPDRS III before LDp/CDp initiation was routinely assessed during wearing-off periods, but timing relative to the previous dopaminergic dose was not systematically standardized. Therefore, changes in MDS–UPDRS III should be interpreted with caution. Similarly, the retrospective design precluded item-level analysis of MDS–UPDRS IV, limiting separate characterization of changes in dyskinesia and OFF time. LEDD calculations were based on standard conversion factors and may not fully reflect individual pharmacodynamic variability, while concomitant medication changes and other confounders were not systematically controlled.

Neuropsychiatric adverse events may have been subject to reporting bias, particularly for mild or transient symptoms, as no structured or blinded assessments were applied and validated scales for psychosis, hallucinations, or impulse control disorders were not systematically used. Follow-up duration was heterogeneous and relatively short in some cases, potentially limiting the detection of late-emerging adverse effects or long-term treatment sustainability. Finally, infusion parameters were highly individualized, reducing comparability across patients.

Prospective, multicenter studies with standardized infusion protocols, systematic neuropsychiatric evaluations, longer follow-up, and patient-centered outcomes are needed to confirm and extend these findings.

## Conclusions

LDp/CDp infusion was generally well-tolerated in this cohort of patients with advanced PD, with neuropsychiatric adverse events being mostly mild, although more frequent than previously reported. Severe psychiatric complications were rare and manageable through an individualized, case-by-case approach. Careful screening, particularly of frontal executive function, together with longitudinal monitoring and personalized management of adverse events, remains essential to optimize treatment safety.

## Data Availability

The data that support the findings of this study are available from the corresponding author upon reasonable request.
